# Family Planning in the Sierra Leone Ebola Outbreak: Women's Proximal and Distal Reasoning

**DOI:** 10.1111/sifp.12210

**Published:** 2022-08-22

**Authors:** Gillian McKay, Luisa Enria, Sara L. Nam, Maseray Fofanah, Suliaman Gbonnie Conteh, Shelley Lees

**Affiliations:** ^1^ Department of Global Health and Development The London School of Hygiene and Tropical Medicine 15‐17 Tavistock Place London WC1H 9SH UK; ^2^ University of Sierra Leone College of Medicine and Allied Health Sciences A. J. Momoh Street Tower Hill Freetown Sierra Leone; ^3^ Independent; ^4^ Independent Consultant

## Abstract

Sierra Leone was highly impacted by the 2014–2016 West Africa Ebola outbreak, with 3,955 recorded deaths. Already stressed maternal health services were deeply affected by the outbreak due to fears of viral transmission, reallocation of maternity staff, and broader policies to stop transmission including travel restrictions. This research sought to explore women's perspectives on delaying pregnancy during the Ebola outbreak using family planning methods. Qualitative data collection took place in Kambia District in 2018 and included 35 women participants, with women who were either family planning users or nonusers at the time of the outbreak. Women reported a variety of reasons for choosing to take or not to take family planning during the outbreak, which we categorized as proximal (directly related to the outbreak) or distal (not directly outbreak related). Proximal reasons to take family planning included to avoid interacting with health care spaces where Ebola could be transmitted, to avoid the economic burden of additional children in a time when economic activities were curtailed and to return to school when education resumed postoutbreak. Distal reasoning included gender roles affecting women's decision making to seek family planning, concerns related to the physiological side effects of family planning, and the economic burden of paying for family planning. Women's perspectives for choosing to take or not take family planning during the Sierra Leone Ebola crisis had not been explored prior to this paper. Using the lens of family planning to consider how women choose to access health care in an outbreak gives us a unique perspective into how all health care interactions are impacted by a generalized outbreak of Ebola, and how outbreak responses struggle to ensure such services remain a priority.

## INTRODUCTION

Amid a global pandemic of Covid‐19, women's ability to seek family planning (FP) services has been negatively impacted across the world due to restrictions in the availability of health services and commodities, out of fear of contagion at clinics, and due to lockdown or movement restrictions (World Health Organization 2020; Cousins 2020; Riley et al. 2020). Crises such as outbreaks and pandemics can cause an increase in unintended pregnancies, making the need for FP all the more acute (Riley et al. 2020). These challenges have been foreshadowed in previous outbreaks, including in the large West African Ebola outbreak that started in 2013 and continued until 2016, where a total of 14,122 people were infected and 3,955 died of the disease (World Health Organization 2016).

The impacts of the West African outbreak of Ebola on reproductive health have been well documented (Sochas, Channon, and Nam 2017; S.A. Jones et al. 2016; Yerger et al. 2020). Following the outbreak there were intensive processes of reflection in numerous “lessons learned” fora into how the tunnel‐visioned focus on stopping transmission of the virus was highly detrimental to the overall health care system, thus impacting on non‐Ebola health services (including reproductive health care). Part of these analyses included how the already weakened health care systems of the three most affected countries (Sierra Leone, Guinea, and Liberia) likely contributed to the extent of the crisis (Shoman, Karafillakis, and Rawaf 2017). Prior to the outbreak, Sierra Leone (the country in which this research took place) had 0.2 physicians and 1.7 nursing or midwifery staff per 10,000 citizens, and only paid 12 dollars per head for health out of the government budget (World Health Organization 2015). McPake and colleagues explain how the fragility of Sierra Leone's postconflict health system, plagued by challenges including poor human resources for health and lack of trust in institutions, likely contributed to the extended outbreak and high death toll (McPake et al. 2015).

The Ebola outbreak, in such a context, can be thought of in terms of syndemics, a “clustering of two or more diseases within a population; the biological, social, and psychological interaction of those diseases; and the large‐scale social forces that precipitate disease clustering in the first place” (Mendenhall 2017). The complexity of accessing maternal health care during Ebola in a syndemic environment meant that while 3,955 people died in Sierra Leone from Ebola itself (World Health Organization 2016), it has been estimated that there were an additional 549–714 maternal deaths due to the weakened health system (Sochas, Channon, and Nam 2017), compounded by the deterioration in trust between women, health providers, and the health system (Nam et al. 2016). These additional maternal deaths were in a country where in 2013, the year before the Ebola outbreak, the Sierra Leone demographic and health survey (DHS) estimated the maternal mortality rate among the highest in the world at 1,100 per 100,000 live births (Statistics Sierra Leone and ICF International 2014).

Pregnant women are particularly vulnerable during Ebola outbreaks (McKay et al. 2019). Pregnancy complications and Ebola symptoms are very difficult to differentiate even by medical experts, and the Ebola‐positive body fluids from complicated deliveries or maternity procedures can cause infection in the health provider especially when personal protective equipment (PPE) is in short supply (Black, Caluwaerts, and Achar 2015; Black 2015). These challenges led many health providers to restrict their work with pregnant women, at least in the early stages of the Ebola outbreak before there was sufficient PPE to allow pregnant women to be cared for safely (S. Jones and Ameh 2015; S. Jones et al. 2017; Strong and Schwartz 2016; Yerger et al. 2020).

The well‐documented and acknowledged risks associated with pregnancy in Sierra Leone during the Ebola outbreak raise the question of why there was not a concerted effort to implement a program to prevent unintended pregnancy, in line with the standard practice in the Minimum Initial Service Package (MISP) for reproductive health care in humanitarian crises (Inter‐Agency Working Group on Reproductive Health in Crisis 2010). While the MISP in 2014 did not include prevention of unintended pregnancy as a lone key objective in crises, this activity was included under Objective 5: Plan for the provision of comprehensive RH services integrated into primary care, and therefore the global policy framework was present to encourage FP service provision. Further to this, the Inter‐Agency Standing Committee reference group for gender in humanitarian action at UN Women issued a gender alert that without increased attention paid to FP services, there was a risk of an increase in unintended pregnancies (2015).

Quantitative analyses demonstrated that while there was a decline in FP service provision, it was less than may have been expected and bounced back to near pre‐Ebola levels quickly (Bietsch, Williamson, and Reeves 2020). Other research has shown that the decrease in service utilization for FP and other reproductive health services was due to a decrease in demand and access issues and less due to supply‐side issues or a decrease in the provision of care (S. Jones and Ameh 2015). These studies however do not explain individual motivations for seeking (or avoiding) FP services during the outbreak.

This paper investigates women's decision making whether to seek out FP services or not during the Ebola outbreak. While we explore women's “individual” decision making, we acknowledge, and will provide evidence in this paper, that women's agency to seek FP in this context is influenced by broader economic realities, political factors, and family dynamics. This paper contributes to the scarcity of evidence on FP in outbreak settings and strives to provide insights that will support demand creation for modern FP in future outbreaks of infectious disease, including the current Covid‐19 pandemic.

## METHODS

All data collection took place in Kambia District, Sierra Leone, where a protracted Ebola outbreak with 286 confirmed Ebola cases occurred from September 2014 to September 2015 (Sandi et al. 2017). Kambia is a rural district, sitting on the border with Guinea with significant migration and circulation for reasons of trade and family ties between the two countries. This district is also the site of other London School of Hygiene and Tropical Medicine projects including the EBOVAC Ebola vaccine studies. This site had a good research infrastructure, including the availability of trained research assistants and a highly engaged Paramount Chief who was widely supportive of research efforts and thus provided local approvals. The fertility rate in Kambia for three years preceding the 2014 DHS was 5.8 children per woman compared to 4.9 for the country, and among all districts, Kambia had the lowest rate of modern methods of contraception use in the country, at 5.4 percent compared to 20.9 percent for the country overall (Statistics Sierra Leone and ICF International 2014).

The primary investigator (GM) on this study had spent time in Kambia in 2014 and 2015 during the Ebola outbreak while working for an international nongovernmental organization (NGO) on a surveillance project, and then returned in 2018 to conduct this research. The main research assistant (MF) was a female in her early thirties, a native‐born Kambian, with language skills in most of the languages spoken in the district (Themne, Susu, Fula, and Krio), as well as English, and worked during the outbreak for NGOs on community engagement projects.

From January to August 2018, a total of 35 women were interviewed for this research, 19 through semistructured interviews and the remaining 16 in two focus group discussions (FGDs). Interviewed women were identified following FP clinics run by a national family planning NGO, or as they were leaving under 5 child health clinics. Focus group women were identified through engagement with local women's groups. Among the interviewees, 10 women had been using modern FP during the Ebola outbreak, and nine had not been. The FGDs were a combination of FP users and nonusers, with one group made up of women from the semiurban area of Kambia town and the other group with women from a more rural area. Women in interviews and FGDs were mixed between married and unmarried, all were between the ages of 18–40 at the time of the interview and identified as either Muslim or Christian. The PI also conducted observations of outreach and static FP clinics and held interviews with health workers about the provision of FP services during Ebola.

The topic guide was developed and piloted in the first few interviews and was adapted over time to further develop themes as they emerged. The FGD topic guide was developed following the interviews to allow the PI to probe areas of interest that had come up in the preliminary analysis of the interviews. During FGDs, the research team employed participatory learning and action tools, including health care journey mapping and a ranking exercise, to help women explain how they prioritized family planning and reproductive health during the outbreak. The analysis took place through free coding of interviews and FGDs using Nvivo 11, followed by a thematic grouping of codes to identify factors that influenced women's decision making to take or not take FP during the Ebola outbreak.

Ethical approval was received from the London School of Hygiene and Tropical Medicine ethics committee and from the Sierra Leone Scientific and Ethical Review board. Local permission was granted from the District Medical Officer and from local traditional authorities (Paramount Chief, local Chiefs, and Village Headpeople). All women were consented in Krio or their local language, were offered the opportunity to ask questions prior to accepting or declining to participate, and were provided with contact details of the PI and local health services should they have questions or need follow‐up support.

## FINDINGS

A lack of availability of FP methods as a barrier to uptake has been documented in Sierra Leone as a key reason why individuals do not use modern pregnancy‐prevention methods (Shirley et al. 2014; Labat et al. 2018). However prior to the start of the outbreak, there had been a significant increase in women using modern FP, from 8 percent to 21 percent from 2008 to 2013 (Statistics Sierra Leone and ICF International 2014), indicating that availability was improving, along with uptake. Observations during this research identified that while FP was meant to be available in static clinic settings, supply chain availability of specific FP methods and trained staff to provide them was not guaranteed. Conversations with health workers identified that FP services had become less available during the Ebola outbreak, as there were reductions in staff numbers and in services offered, which is backed up by research showing an FP distribution decrease of 23 percent (Bietsch, Williamson, and Reeves 2020). Outreach services provided by a national family planning NGO were better supplied with FP commodities so offered a greater range of methods, but staff from the NGO reported that these services were also reduced during the outbreak for staff safety reasons.

In setting the scene in the FGDs, the research team asked women to free list and rank their concerns during the outbreak, to better elicit where concerns around pregnancy and FP would fall. The findings from these exercises identified that the top concerns of women were not directly health related, with main issues such as a lack of prayer and group gatherings (including funerals) being banned, schooling for children being stopped, and trading and other money‐generating activities being impacted. Some of these issues can be indirectly linked to health, as despite efforts by the Sierra Leone government to provide free health care as part of the Free Health Care Initiative for pregnant and lactating women and children under aged 5, informal payments are still often required at the point of care (Pieterse and Lodge 2015; Witter et al. 2016) meaning the inability to earn money would make seeking health care more difficult. Concerns related to fear of catching Ebola were mentioned and ranked highly, as women were concerned about being quarantined for possible Ebola exposure, and the subsequent economic impacts on their livelihoods. While pregnancy and FP were not the most critical concerns of the women in our FGDs, these issues did come up without prompting and were said to be linked to not wanting to get pregnant during the outbreak, due to their fear of attending health facilities.

## PROXIMITY TO DISEASE

Many of the factors that women reported have impact on their decisions to take (or not take) FP related to the proximity to the disease either in the form of people (like health care workers) or geographic locations (like health care centers). This is a similar idea to that described by Shrum et al. when discussing Ebola across many countries, who used the term “locative,” which they defined “as concern for one's personal well‐being in spaces where microbial threats are, have been or might be*”* (Shrum et al. 2020). This framework is particularly useful in thinking about Ebola given how it is transmitted, through contact with bodies or body fluids, and the role of health care facilities in the potential mitigation or spread of the infection.

### Taking FP to Mitigate the Risk of Ebola

The case definition in 2014 for Ebola included unexplained bleeding, vomiting, diarrhea, and other symptoms that could be confused with early pregnancy symptoms, and symptoms of complications of pregnancy (Black 2015). This was of concern to participants who believed it could lead to women being identified as suspect Ebola cases and being sent to an Ebola treatment center, far from their homes and families, possibly not to return.

Because the signs and symptoms of pregnancy are similar to Ebola. Some women do get sick from one month to five months when they are pregnant. So, during Ebola when one vomits they will just call 117^1^ for you, and they come and take you the Ambulance will again say good bye by saying “owa‐o” owa‐o! owa‐o^2^!. FGD with rural women

Fear of the potential negative outcomes of pregnancy and delivery was a large driver for many women to go on or continue with FP. The perception of poor care being provided at the health center was common, many women told stories of friends and family members who had been treated badly or died while delivering their babies, or who had been taken away to an Ebola treatment center and had never returned.

Because during the Ebola if you get pregnant you will not get care because at any time you visit the hospital the nurses are scared to touch you, the people will think you are an Ebola patient. That is why we are scared to [go to the hospital]. FP user, 18–25 years old

Even women who had not taken FP during Ebola felt that these concerns justified the taking of FP and therefore advised others to take FP.

I will advise [my daughters] to take it because I will make reference to that pregnant woman who was vomiting and when they carried her [to the Ebola treatment center] she did not [return to her family]. FP nonuser, 26–40 years old

### Contagion and Distrust Leading to the Avoidance of FP Spaces and Providers

Seeking out FP by going to the clinic was believed to put women at risk for catching Ebola. Therefore, some women felt it was better to see health workers privately, at the provider's home or outside the clinic setting.

Our people were not allowing us to go to the hospital and they always advise us that when you go to the hospital they will do this or do that [test you for Ebola]. Because of that, we will wait until the area nurse is off from work then we go there [to their home] and take [FP]. FP user, 18–25 years old

In some cases, it was the health workers who were perceived to be carrying or spreading the infection, thus they were to be feared or avoided.

“As for me, my neighbour is a nurse and was working at the centre, when she came home and washed, she will call me to play [a game], I always said no. So, at one time some spots began to appear on her face, I became afraid of her, when she comes and sit down I will go inside and sleep. After the Ebola she was laughing at me saying that she noticed that I was afraid of her.” FGD with urban women

They were afraid of the nurses, because some people do not even believe/trust the nurses… and people were afraid of Ebola and because we heard that a lot of health workers died during Ebola. FP nonuser, 18–25 years old

This distrust of health workers also manifested in relation to what FP method was considered safe to take. Prior to Ebola, the most commonly used method was injectable contraception (Bietsch, Williamson, and Reeves 2020). However for some women, this method was no longer acceptable, as there were persistent rumors that health workers used injections to give people Ebola (S. Jones et al. 2017; Dynes et al. 2015).

Because during that time we were afraid, we felt they were giving Ebola [injections] or if you go to seek prevention^3^, you might not know the person who is treating you, they might give you another injection that is not prevention, so that was why we were afraid during that time. FGD with urban women

One way for women to overcome this concern about injections was by choosing FP methods that did not require physical contact between the health provider and the woman's physical body.

For me, I prefer the pills because it will not give reason for somebody to touch me like it happens in the case of the injection. When I buy [pills], I will go to my house and take and nobody will touch me to give me injection at that time. FP user, 18–25 years old

The challenges women faced in trusting their health providers were often related to PPE. PPE at the primary health care level was not the head to toe, anonymizing, yellow PPE worn in Ebola treatment centers , and yet it was still fear inducing for many women:

That is why some are afraid to go to the centre because of the PPE…because when they wear the PPE is like a ghost, even if you know someone, when they wear the PPE, you will not recognize the person. FGD with rural women

### Overcoming Fears through Interpersonal Relationships and Confidence in Known Health Workers

PPE could however contribute to increasing trust between a health provider and some women when they sought out FP or other clinical care because in the “no‐touch environment^4^” PPE allowed for greater physical contact between patient and health care worker and induced a sense of reassurance.

I felt good because since they said we should not touch and the protective gears she put on will help to protect herself and me because we both did not know our status at the time. That's why I felt good because they put on their PPEs. FP user, 18–25 years old

While proximate fears of contagion from health workers could lead to women choosing not to take FP, these fears could be overcome with a sufficient level of trust and engagement with health workers from their local area, especially if these workers spoke the same language and came from the same geography.

Like, I got used to so many nurses in the community because some of them are native born of the land. If I am a native born of [town] and they brought me here as a health worker, if my sisters see me they will have confidence to go take the prevention [FP]. FGD with rural women

This familiarity with the health worker may have also helped to overcome women's fears of PPE, as the worker is less anonymous even when masked and gowned when they are a person the woman already knows.

### Concerns about Side Effects of FP Mimicking Ebola Symptoms

How women saw their bodies during Ebola may have had an effect on their willingness to take FP. Common side effects of FP (like breakthrough bleeding) could be considered a signal indicator of potential Ebola infection and may have caused some women to rethink taking FP, out of concern that they could end up in an Ebola treatment center.

It is because some women, when they take prevention they will bleed too much, but during Ebola when you bleed they will say it is Ebola, and some say they get stomach pain and during Ebola even if your stomach ached they will call 117 for you and if you go [to the Ebola treatment center], you will not come home again. FGD with rural women

### Abstinence as a Way to Prevent Ebola

A number of women stated that they did not need to use FP as they chose to limit sexual contact with their partner in order to comply with the emphasized message of “no‐touch” to prevent Ebola transmission. Further to this, sexual transmission of Ebola has been documented (Schindell, Webb, and Kindrachuk 2018), and this risk was well publicized during the Ebola outbreak with Ebola survivors given supplies of condoms on discharge from an Ebola treatment center.

The reason why people were afraid of “mami en daddy bisnes” [sex], why I was afraid of sex, because they said during the Ebola, we should not touch one another. So, my husband used to go out to work, so I might not know if he met with somebody who has Ebola and then he comes and touches me, so there was that fear, so I did not allow him. FGD with urban women

But some of the boyfriends now, they can leave you and have another woman, so you might not know if the person he is going out with is sick, so that is at the time when Ebola came, I closed my door on all of my boyfriends, I didn't have boyfriends again, I was afraid. FGD with urban women

Some women interviewed, located in a village that had been entirely quarantined near the end of the Ebola outbreak, spoke about how they chose to completely avoid sexual relations during the 21‐day quarantine period. The Ebola narrative in this village was that a woman who passed away from Ebola had contracted it from sexual contact with a known male survivor, leading to many women in the village refusing to sleep in the same beds as their husbands. The agency that these women were able to show is in contravention of typical gender norms in Sierra Leone (see below section on gender relations) with regard to who holds the sexual power in couples (almost always the men) in Sierra Leone (Fofana Ibrahim 2017) and demonstrated the exceptionality of Ebola.

### Preventing Economic Burden through FP

The additional stresses of the impact of Ebola on the economy were a driver for some women to take FP or to prevent pregnancy through other means including abstinence. Many markets were closed restricting petty trading, and large mining companies also closed, putting many people out of work and plunging them into economic hardship.

… when there is no money and you have to go to clinic it is difficult. Even if they say free health care^5^ , you [pay a small amount]. There are some medicines that are not under free health care, you have to buy them. So, if you are pregnant during [Ebola] you have to spend money. So, when you give birth to the baby you have buy things for the baby, so that is why when there is no money child bearing is not sweet. FGD with urban women

The impacts of the economic decline due to Ebola among women were more severe than among men due to women's higher presence in highly Ebola‐impacted sectors including markets, cross‐border trading, hospitality, and farming (African Development Bank 2016).

### Attaining Educational Goals through FP Despite Ebola

To stop or restrict transmission during the Ebola outbreak, the government closed all public and private schools for a year. The closure of schools was a motivating factor for many women to take up or continue with FP, as they did not want to become pregnant during the school closure as they could have difficulty completing their schooling or exams when they reopened. The following respondent had a baby at the age of 15, causing her to leave school early but she returned to finish her studies before the outbreak began. In this quote, she explains how FP was important to her to enable her to continue her studies.

No, I was never afraid [to take FP]. The moment it got expired I will immediately go again and take after my menses … I was preparing to take my [national examination]. The schools were closed but I was taking extra lessons, so you know when schools reopened I could go back. There were rumors that schools were about to reopen so I was taking extra lessons to catch up so that when the schools reopened, I would have got some good preparation. FP user, 26–40 years old

### Choosing to Have Children amid the Ebola Outbreak

Decision making was for many women related to their own personal circumstances and their support networks that could facilitate having a first or additional child. While most women did not wish to become pregnant during the Ebola outbreak, other women did desire to have more children, and this was the major factor in their decision not to take FP. “Because my husband had no child at that time that is why I decided to get a child for him. I wanted also to have a baby. I did not have fear about having a baby during Ebola time” (FP nonuser, 18–25 years old).

Fertility intentions have been studied in other crises, and it appears the type of crisis may contribute to women's desire to have future children. Following the Angolan war, women in less‐affected areas were more likely to wish for a pregnancy, compared to women in more‐affected areas (Agadjanian and Prata 2002). During the Zika outbreak in Brazil, in areas with more cases of microcephaly (the birth defect caused by in utero exposure to Zika virus), there was a corresponding decrease in childbearing, indicating women were preventing pregnancy (Diaz‐Quijano, Pelissari, and Chiavegatto Filho 2018). Following the 2004 Indian Ocean Tsunami, women in areas with higher mortality were more likely to have additional children than in areas that were less affected (Nobles, Frankenberg, and Thomas 2015).

## DISTAL TO DISEASE BUT STILL AFFECTED BY EBOLA

Outside of the proximate reasoning that drove women to choose to take or not take FP, there were also additional reasons that emerged from this research. These reasons are not as “Ebola‐specific” in that they are chronic challenges that women face, but were amplified during the Ebola crisis. We have termed these “distal” as while they are further away from the locus of the virus itself and the concerns around contagion, these reasons are still affected by the context of the Ebola outbreak.

### Gender Roles Determining Choice to Take or Not Take FP

For many women, the unequal gender relations common in Sierra Leone were highly influential in their decisions either to take or not to take FP. These challenges were not unique to the Ebola context, but instead they highlight the background against which Ebola compounded preexisting, often structural, challenges to seeking FP.

For some women, their reasoning to avoid future pregnancy was due to having unreliable partners, who would not be supportive of them or their children. Choosing to take FP was because “men of this generation are not serious” (FP user 18–25 years old) or that the men were in relationships with other women, or that they would not care for the baby “my husband does not care about me and the child” (FP user, 18–25 years old). Women's role as the primary caregivers for children and as the member of the couple responsible for obtaining FP (when the man has no responsibility to wear a condom) has been further discussed in research by Fofana Ibrahim (2017).

Other family pressures could also work against a woman choosing to take FP, if, for example, her husband did not want her to be on a method to control her sexuality.

One of my friends told her husband that she wanted to go and take prevention, her husband said no, because she wants to prostitute that is why she wants to go and prevent. So, they quarreled. FGD with urban women

The control that men have over women's sexuality is in contrast to how men were able to go about their sexual lives, even during Ebola. Fofana Ibrahim describes a case of a woman who caught Ebola following sex with her husband. She believed he had been in an Ebola treatment center, but he told her that he had simply been living with his other wife in another town, and when she challenged him he became angry and threatened to leave her, with no financial support, so she complied and had unprotected sex resulting in her illness (Fofana Ibrahim 2017).

### Concerns Related to Potential Physiological Effects of FP

Many women interviewed had concerns about FP side effects. Concerns about changes in menstruation, either excess or reduced bleeding, were common. While this was made more acute during the outbreak out of fear of being sent away to an Ebola treatment center if the bleeding was thought to be “unexplained,” the side effects alone were also reason enough to avoid FP.

When I was taking family planning, it was stopping my menses and it caused me daily abdominal pain. So after several struggles with it, I eventually experienced shortness of blood and for that reason, I had to remove it. FP user, 18–25 years old

Other women recounted stories of side effects that their friends or family members had perceived they had suffered due to FP. While it must be noted that these are not biologically supported side effects, these do reveal real anxieties and the importance of prior narratives and experiences of women or others that influence decision making.

I decided not take family planning because my Aunty had taken family planning when she was young, she took an implant. So, when the war came, she was not able to go to the hospital again for them to remove it, so the implant remained inside her and it later turned cancer, and stated eating into her body and she died of that. FGD with urban women

Why some women do not take this prevention is that they say when they take it, it blocked their womb not to give birth and that they will never give birth again. FGD with urban women

This concern over side effects also came into play when women were discussing the potential impact on children, namely on babies still breastfeeding as one woman who was not on FP during Ebola (but who later took injectable FP) stated “They said the prevention [FP method] will affect the child” (FP nonuser, 18–25 years).

### Economic Burdens Factoring into Decisions to Take or Not Take FP

While FP is meant to be free in Sierra Leone, many women reported having to pay at the public health unit or at a private clinic. This has been documented previously and often stems from the lack of a “whole of the health system” approach to resourcing such an initiative, where health workers salaries are not guaranteed, leaving patients to pick up the costs, and where overworked staff are thought to provide poorer care especially in rural areas (Witter, Wurie, and Bertone 2016; Pieterse and Lodge 2015; Wurie, Samai, and Witter 2016). The amount women reported having to pay ranged from 5,000 to 10,000 Sierra Leone Leones (approximately 1–2 USD at the time of interview). The financial cost of getting FP for some women did factor heavily into their decisions, as the reported cost could represent an entire day's earnings, or even more if the woman's earnings had been reduced due to Ebola's impact on the economy.

I stopped taking injection before Ebola because I hadn't money to pay and by the time I got the money, I was already pregnant with my first child, but it was miscarried and it was during the Ebola. FP nonuser, 18–25 years old

Although economic hardship was also in some cases a driver for women to take FP, if they perceived that times were difficult and therefore it would be better not to have another child.

It's all about hardship, my child has to go to school and I have nothing, I have to dress him, I have to please him. If I have plenty of children and things are difficult, it will be difficult to meet their demands and it will lead them to the street to beg. That is why I gave birth to the number I can care for. FP user, 18–25 years old

Again, these economic burdens were increased during the Ebola period; however, the quotes above represent the reality that for many women, regardless of the crisis, economic reasoning is critical in their decision‐making process around FP.

## DISCUSSION

Women's individual decisions and agency that enabled them to choose to take or not take FP during the Sierra Leone Ebola crisis had not been explored prior to this paper. These findings show the significance of proximal and distal reasons for choosing or choosing not to take FP, and that, while difficult to differentiate at times, looking at both types of reasons demonstrates a holistic view of how FP care seeking was disrupted or adapted to in the outbreak. Using proximal and distal framing allows for the impacts directly related to the virus (proximal) to be differentiated from the chronic and structural challenges of seeking gendered health care in a country with a stressed health system such as Sierra Leone (distal). Furthermore, the framing of distal allows us to keep sight of how while these reasons may have preceded the outbreak, the outbreak itself added additional pressures to health and social systems, making these challenges more acute. Using the lens of FP to consider how women choose to access health care in an outbreak gives us a unique perspective into how all health care interactions are impacted by a generalized outbreak.

Proximal fears including concerns around hygiene and contagiousness of staff directly impacted on women's decision making to seek services. Hospitals and health facilities are meant to be places of cleanliness, healing, and safety, yet even in “normal” or nonoutbreak times they can be sites of contamination and disease (Nejad et al. 2011; Abdullah and Kamara 2017). However, the Ebola outbreak amplified any preexisting concerns women may have had about catching a disease from a health facility setting, possibly due to the overwhelming messaging from government, NGOs, and the overall Ebola response about the importance of handwashing and “no‐touch” in the prevention of Ebola.

On a physical level, women's fears and concerns about how their own bodies could be seen as sources of contamination or disease by health workers, especially in cases of “unexplained bleeding” during menstruation or due to side effects of many FP methods, were legitimate given a context with broader narratives of people being sent away to an Ebola treatment center. While “unexplained bleeding” was not restricted to blood from the womb, as any kind of bleeding was potentially an Ebola sign, women and girls are unique in that they bleed regularly, and while this should be seen as “explained” physiological bleeding, menstrual blood is widely seen as a contaminant in many societies (Tan, Haththotuwa, and Fraser 2017; Hoskins 2002). The negative connotations of menstrual and FP side effect blood may thus have been (inappropriately) aligned with blood resulting from a spontaneous miscarriage, of which Ebola infection can be an inciting factor (Black 2015). Mary Douglas’ concept of “matter out of place,” the idea that blood should be on the inside, applies here (Douglas 2003). As menstruation implies “bleeding without injury” this bodily function could be misinterpreted by health workers as an Ebola sign that would justify sending a woman away to an Ebola treatment center, reinterpreting a normal bodily process as one that is threatening. This disruption of the body's normal processes is mirrored in the societal disruption of Ebola time, leading women to possibly consider their bodies as abnormal or contaminated, to be feared or as a source of disease and infection to their loved ones.

This fear of women infecting health workers with Ebola was mirrored with women's fears about health workers as a potential source of contamination. This fear, however, could be mitigated (though not eliminated, given how the nature of the outbreak response negatively impacted on confidence and trust in health services) through a social connection with the worker themselves, helping to increase trust in safe care. Abstractly women reported that health workers could infect them, either intentionally through injection with Ebola (as they perhaps had heard through circulating rumors) or unintentionally through unsafe care. On a more personal level, women acknowledged that when they knew the worker and/or when the worker was from their local area, they were more able to trust them and this abstracted fear was reduced. The importance of having trusted health workers delivering services during an outbreak cannot be understated, especially when there are increased fears related to unfamiliar practices like the wearing of PPE, concerns around Ebola‐injections and a generalized environment of suspicion and rumor. However, it must be acknowledged that this trust can be misplaced in cases where a health worker is in fact ill, is not well trained and equipped with PPE, and continues to provide care, thus infecting their patients, as was sadly seen in some cases during the outbreak (Manguvo and Mafuvadze 2015).

Building rapport and developing familiarity with health workers has been shown in other studies of the Sierra Leone Ebola outbreak to increase trust even in potentially risky situations. For example, Ronse's research identified trust in the national and international trial staff and a sense of social responsibility as key in the decision of Ebola survivors to be plasma donors in the search for a treatment for the disease (Ronse et al. 2018). Additionally, Enria and Lees (2018) found that participants agreed to take part in an Ebola vaccine trial when they felt connected to the staff of the trial, either personally or through close social networks.

The factors influencing decisions around whether to choose to become pregnant or avoid pregnancy during an outbreak are complex and touch on both proximal and distal reasoning. Some women had to overcome concerns of contamination, family and partner pressures, and economics. For others, the outbreak was simply not a large factor in their decision making, possibly due to the long period in which the outbreak took place, from approximately March 2014–June 2016. For these women, life had to go on, it could not be put on hold indefinitely waiting for the virus to be overcome, a finding also described in Lipton's (2019) chapter about pregnancy in Freetown during the outbreak. As discussed by Vigh in his 2008 paper about crisis and chronicity, for those who live in unstable contexts where crises are endemic, the external framing by crisis responders is that these huge upheavals in society will change the lives of all, yet for individuals living through the crisis, they may see it as just one more event that must be adapted to (Vigh 2008). Outbreak responders tend to see the crisis as all encompassing, not recognizing that for many people, the chronic concerns of daily life, including challenges and difficulties in accessing health care (whether economic, social, or physical), have been amplified by the outbreak, and so efforts must be made to reduce additional barriers that have been created due to outbreak response policies.

Some women were able to, in a way, take advantage of the Ebola situation to assert a different form of power in the household in relation to their reproductive and sexual decision making in contrast to findings by Fofana Ibrahim (2017) that women had no choice with regard to sex with their partners. In this research, some respondents reported being able to tell their partners that they were not available for sex due to the no‐touch policy and their concerns about partners bringing Ebola into the home. In this way, they asserted their rights and authority using pragmatic means mid‐outbreak. How their partners responded to this was not investigated in this research but it was the older women in the FGDs and KIIs who described such powerful interactions, implying a different family dynamic than may exist among younger women. This pragmatism also applied to women who chose to seek out FP, in spite of concerns about potential contamination, out of their desires to complete schooling or maintain some economic stability in a time of intense economic disruption. As one respondent stated in an assessment from the 2018–2020 Ebola outbreak in the Democratic Republic of the Congo, “Ebola‐time is a good time to plan your family,” further evidencing that women and their partners should be offered the opportunity to prevent conception if they so desire, to assert agency over their lives during and after the crisis (McKay et al. 2019).

Unfortunately, amid highly complex public health crises like Covid‐19 and Ebola, FP often becomes considered a nonessential service as health workers are reassigned and service availability is restricted for safety and capacity reasons. A 2020 survey on essential services in Covid‐19 found that 68 percent of countries reported a partial or severe disruption in FP (World Health Organization 2020). The rationale for the nonessentialness of FP is likely due to the service being considered (by some in the humanitarian space) as part of development and long‐term programming, not part of humanitarian or emergency programming. There are constant tensions when development actions (including FP) cease or are deprioritized during a humanitarian crisis, to the detriment of those who depend on such services and may even need these services more during the crisis itself given social upheavals. This mismatch in perspectives between the lived experience and needs of women in the Ebola outbreak, and the response structure's ability to identify, integrate, and respond to these needs demonstrates how emergency responses, like outbreaks, struggle to address health problems that are not directly outbreak related, especially in fragile health systems.

It is not possible to separate the long‐term and chronic needs that women have for quality reproductive health services (especially in a state of health system fragility) from the acute challenges that are amplified during a public health emergency, for example, when health workers are reassigned away from reproductive health areas, thus reducing service availability. These issues reflect ongoing conversations, and academic critiques, of the humanitarian‐development nexus, wherein the humanitarian principles of immediate and needs‐based response are at odds with an approach focused on long‐term, rights‐based development. Expanding the “humanitarian present” (the immediacy of the response environment) to encompass preventive and recovery stages aims to bring together humanitarianism and development, aiming to reduce the disruptions of crises on populations (Lie 2020). These ideas, if they can be successfully implemented (which is an ongoing challenge) would acknowledge and react to the reality that women's decision making is not done purely in the moment in a crisis, as the long‐standing impacts of the context in which she lives will inevitably impact on her choices of how, from who, and where to seek care. Real, practical actions to reduce the chasm between humanitarian and development are not always easy to identify, but FP advocacy to ensure that FP services are ring‐fenced in current and future outbreak responses, as has been argued in a paper about essential services in Covid‐19 (Blanchet et al. 2020), could be a step in the right direction.

## LIMITATIONS

There are several limitations to this research. First, by recruiting women outside health clinics we may have biased the sample towards women who were more likely to be engaged with health services (including FP). Some women declined to participate in the interviews but we did not record their reasons for nonparticipation, so it is not possible to know how this may have impacted the sample. Second, the relatively small sample of participants across rural and more urban environments meant it was not possible to identify particular barriers or opportunities unique to those environments. A final limitation may be that by asking women to provide information about their experiences from several years prior that they may have struggled to recall their decisions or challenges. Though in general it appeared that the Ebola outbreak was a major event for many of the women interviewed, and thus they had vividly recalled stories to share, even if occasionally they were not able to remember the dates of the events they described.

It is difficult to say if similar findings would be found in other areas of Sierra Leone. The extent of widespread Ebola transmission varied across the country, with some regions experiencing much less, and others much more. Women in areas with less transmission may have not experienced quite as much disruption to their health care access, though some level of disruption is to be assumed given national policies related to the Ebola case definition and PPE. Kambia district, even in urban areas, is still quite a rural environment, and thus the findings may be less generalizable to highly urban spaces, like the capital city Freetown, where the trust in health workers may be different, as the local health clinic staff may not be as well known to those frequenting it.

## CONCLUSION

In the midst of the widespread Ebola outbreak in Sierra Leone, policies to stop transmission of the virus and fears of contamination impacted on health care seeking, including for non‐Ebola care like FP. All interactions with the health care system were touched by the outbreak itself, from the process of deciding to seek care when contamination was possible (and negotiating with family to do so), to the reporting of Ebola‐associated symptoms (like vaginal bleeding), to the choice of method, to the confidence to engage with a health worker. While some facets of health care interactions are more proximal than others (e.g., fears of contamination), even distal interactions (like the economics of paying for FP) were amplified by the Ebola outbreak. Women's perspectives of engagement with the health care system in this time of crisis demonstrate how they married their previous experiences with the new environment of Ebola to decide how best to manage their reproductive lives. The Ebola response's ability to engage with women's needs at this time was lacking, and generally added further barriers to care seeking, fueling the critique of health responses divorced from the day‐to‐day lived reality.

## Data Availability

The data that support the findings of this study may be made available on request from the corresponding author. The data are not publicly available for research participant privacy reasons.

## Footnotes

1 The Ebola alert line that could be used to report possible Ebola cases.

2 The ambulance that takes you away makes a noise like “owa‐o,” which also means “goodbye” in Themne.

3 A commonly used term to mean contraception.

4 A key message in Ebola‐time was “no‐touch,” intended to reduce physical contact between people to prevent transmission of Ebola.

5 Sierra Leone Free Health Care Initiative.
